# Anisotropic Cryostructured Collagen Scaffolds for Efficient Delivery of RhBMP–2 and Enhanced Bone Regeneration

**DOI:** 10.3390/ma12193105

**Published:** 2019-09-24

**Authors:** Kai Stuckensen, José M. Lamo-Espinosa, Emma Muiños-López, Purificación Ripalda-Cemboráin, Tania López-Martínez, Elena Iglesias, Gloria Abizanda, Ion Andreu, María Flandes-Iparraguirre, Juan Pons-Villanueva, Reyes Elizalde, Joachim Nickel, Andrea Ewald, Uwe Gbureck, Felipe Prósper, Jürgen Groll, Froilán Granero-Moltó

**Affiliations:** 1 Department for Functional Materials in Medicine and Dentistry and Bavarian Polymer Institute, University of Würzburg, D-97070 Würzburg, Germany; 2 Department of Orthopaedic Surgery and Traumatology, Clínica Universidad de Navarra, 31008 Pamplona, Spain; 3 Cell Therapy Area. Clínica Universidad de Navarra, 31008 Pamplona, Spain; 4 Department of Materials CEIT-TECNUN, Universidad de Navarra, 20018 San Sebastian, Spain; 5 Department Tissue Engineering and Regenerative Medicine, University Hospital Würzburg, D-97070 Würzburg, Germany; 6 Department of Haematology, Clínica Universidad de Navarra, 31008 Pamplona, Spain

**Keywords:** rhBMP–2, collagen sponge, cryostructured scaffolds, bone critical size defect

## Abstract

In the treatment of bone non-unions, an alternative to bone autografts is the use of bone morphogenetic proteins (BMPs), e.g., BMP–2, BMP–7, with powerful osteoinductive and osteogenic properties. In clinical settings, these osteogenic factors are applied using absorbable collagen sponges for local controlled delivery. Major side effects of this strategy are derived from the supraphysiological doses of BMPs needed, which may induce ectopic bone formation, chronic inflammation, and excessive bone resorption. In order to increase the efficiency of the delivered BMPs, we designed cryostructured collagen scaffolds functionalized with hydroxyapatite, mimicking the structure of cortical bone (aligned porosity, anisotropic) or trabecular bone (random distributed porosity, isotropic). We hypothesize that an anisotropic structure would enhance the osteoconductive properties of the scaffolds by increasing the regenerative performance of the provided rhBMP–2. In vitro, both scaffolds presented similar mechanical properties, rhBMP–2 retention and delivery capacity, as well as scaffold degradation time. In vivo, anisotropic scaffolds demonstrated better bone regeneration capabilities in a rat femoral critical-size defect model by increasing the defect bridging. In conclusion, anisotropic cryostructured collagen scaffolds improve bone regeneration by increasing the efficiency of rhBMP–2 mediated bone healing.

## 1. Introduction

Bone tissue is composed of specialized cells (osteoblasts and osteocytes) embedded in an extracellular matrix, which represents a hierarchical network assembled from two major components, collagen type I fibrils and hydroxyapatite nanocrystals, distributed along the collagen fibrils [1]. While the cortical bone bears an anisotropic microstructure, the trabecular bone features isotropic structural properties [2].

Failure of the reparative process of bone tissue is a major clinical problem. Fracture type, anatomical location of the fractured bone, and associated comorbidities of the patient may interfere with the normal process of repair, resulting in fracture nonunion, delayed union, or malunion. Fracture nonunion is a cause of chronic pain and disability, while being associated with increased healthcare cost and loss of working days [3]. The gold standard for the treatment of non-unions is based on the use of bone autografts, but supply is limited and allograft substitutes lack osteoinductive and osteogenic properties [4]. 

Bone morphogenic protein 2 (BMP–2) is member of the transforming growth factor superfamily, with a recognized role in bone and cartilage formation, as well as in fracture healing and repair [5,6]. Due to its osteogenic and osteoinductive properties, BMP–2 is commonly used as an alternative to bone autografts [7,8]. Unfortunately, its short half-life requires that supra-physiological doses are used for efficacy [9]. Besides other attempts, BMP-based treatments require a local and controlled release and are commonly delivered through absorbable collagen sponges [10,11]. Thus, by using special scaffolds, the efficiency of existing BMP–2 treatments might be increased, since the conventional leaching out of loaded BMP–2 could be avoided. 

Additionally, important aspects to consider for the clinical use of BMP–2 are represented by the documented side effects: inflammation, ectopic bone formation, and increased bone resorption at the application site [12]. These adverse side effects can be life-threatening when BMPs are used in spinal cord surgeries [13]. Therefore, dose, carrier, and co-delivery of other growth factors have been investigated in order to increase their efficiency and reduce the occurrence of side effects [14,15,16,17,18].

Anisotropic materials present interesting properties compared to isotropic ones in different aspects of tissue regeneration. In some tissues, i.e., cartilage, anisotropic scaffolds enable guided cell migration by mimicking the layered native morphology [19]. Meanwhile, for controlled release of drugs and/or protein factors, structural anisotropy allows slow release of bioactive molecules or trophic factors [20]. 

In order to investigate valuable structural attributes for bone regeneration, we produced two kinds of scaffolds out of equal weight amounts of murine type I collagen and pre-precipitated sub-micron hydroxyapatite crystals by application of a recently developed cryostructuring-based processing route [19]. These were bone scaffolds with anisotropic channel pores, which propagate from one basis of the cylindrical samples to the other (ANI), and scaffolds with an isotropic, sponge-like pore structure, which are randomly distributed among the matrix (ISO). In this way, the structural morphologies of native bone tissue are matched, isotropic scaffolds recapitulating the structure of spongy bone tissue and anisotropic scaffolds resembling cortical bone. Furthermore, an oriented consecutive porosity could yield favorable results after implantation, due to facilitation of osteoprogenitor cells migration and slower human recombinant bone morphogenetic protein (rhBMP–2) release. The scaffolds were loaded with rhBMP–2 and applied to evaluate the healing of critical size defects in the femurs of Sprague–Dawley rats.

## 2. Materials and Methods 

The rhBMP–2 was produced inside modified *Escherichia coli* bacteria and purified according to previously published protocols [21]. For the synthesis of hydroxyapatite, 0.05 M (NH_4_)_2_HPO_4_ and 0.03 M Ca(NO_3_)_2_·4H_2_O solutions were used, according to a previous described protocol [22]. Collagen type III and type I was isolated from murine tail tendon. Considering the dominant collagenous amounts within these mixtures, it is referred to as “collagen I”. The tendons were disinfected twice for 10 min in 70% ethanol and washed under stirring in 0.9% NaCl solution for 12 h. The dissolution of the collagenous material was carried out by acidic fractioning (0.1% acetic acid) with 100 mL per gram of tendon material. The acidic fractioning was carried out using a RML6 cryostat (Lauda; Lauda-Königshofen, Germany), cooled by a 1:1 ethylene glycol water mixture coupled to a glass reactor. Inside the reactor, tendons were fractured for 28 days at 5 °C under stirring with an E60 KPG stirrer (Heidolph; Schwabach, Germany) at 15 rpm. Finally, the collagen I was lyophilized and stored at −20 °C.

### 2.1. Production of Cryostructured Bone Scaffolds

For the production of cryostructured bone scaffolds, a collagenous suspension was prepared by mixing hydroxyapatite and collagen I, 3.8% each in weight, in 0.5 M acetic acid. The suspension was degassed by centrifugation. A custom built adjustable cryostructuring device was used to create bone scaffolds by controlled ice crystal growth. The anisotropic scaffolds (ANI) were cryostructured with an external temperature gradient of $\vec{\nabla }T$ = (4.750 ± 0.035) K/mm. At a linear interpolated cooling rate of v_c_ = (−0.806 ± 0.093) µm/min, the ice front was propagated with a linear solidification velocity of v_s_ = (505 ± 30) µm/min. The isotropic scaffolds (ISO) were subjected to an external temperature gradient of $\vec{\nabla }T$ = (1.1280 ± 0.0357) K/mm, and a linear interpolated cooling rate of v_c_ = (−0.393 ± 0.043) µm/min, which led to a linear interpolated solidification velocity of v_s_ = (476 ± 27) µm/min (Supplemental Figure S1). After solidification, the specimen was transferred to an Alpha 1-2 LD lyophilizer (Christ; Haan, Germany) where sublimation of the frozen solvent was carried out for 18 h, at −52 °C and 70 µbar.

The resulting porous structure was further covalently cross-linked with a carbodiimide solution containing 3/5 H_2_O, 2/5 Ethanol, 200 mM MES, 52 mM EDC, and 21 mM NHS. Therefore, the structure was placed in a pressure vessel, which was evacuated until reaching an operating pressure of 100 mbar. One hundred µL of the cross-linking solution was added per mg of specimen, and 45 s after the infiltration of the material, the pressure vessel was vented. After 20 h reaction time, the scaffolds were washed three times in distilled water. Afterwards, the scaffolds were freeze-dried again to yield cylindrical samples of 4 mm diameter and 6 mm height. 

The degradation behavior of the bone scaffolds was monitored in vitro for 30 days at 37 °C with continuous shaking. Bone scaffolds were subjected to lixiviation in PBS (137 mM NaCl, 2.7 mM KCl, 7 mM Na_2_HPO_4_, 1.5 mM KH_2_PO_4_) containing 3 mM sodium azide. Five cylindrical scaffolds of each type (4 mm diameter; 6 mm height) were lixiviated in 96 well plates, where 0.5 mL buffer was exchanged in a two-day interval. The initial dry, the wet, and the drained weight of the gamma-sterilized scaffolds were used to determine mass loss, free water content, and equilibrium water content. Associated errors are represented by the respective standard deviations. 

### 2.2. Scaffold Characterization by Scanning Electron Microscope (SEM) and Energy Dispersive X-Ray Spectroscopy (EDX).

In order to prepare cross-section surfaces for the electron microscopy analysis, a cryostatic sectioning was performed at 1 µm thick sections using a Cryostat MNT cryomicrotome (SLEE, Mainz, Germany). An Auriga 60 SEM (Zeiss; Munich, Germany) was used for recording high-resolution images. The samples were thinly coated with carbon and platinum before they were scanned with 3 kV acceleration voltage. Energy dispersive X-ray spectroscopy (EDX) measurements of characteristic sample areas were performed within the Auriga 60 SEM using an Apollo XL SDD detector. The EDX spectra were captured with 5 kV acceleration voltage with a resolution of 133 eV. 

### 2.3. Scaffold Mechanical Testing

The mechanical testing measurements for the collagen sponges were carried out in PBS at 25 °C using an Eletroforce 3220 (Bose GmbH, Friedrichsdorf, Germany) with Wintest 4.1 software (BOSE GmbH, Friedrichsdorf, Germany). The scaffolds were tested in elastic response inside a custom build confined compression setup (4 mm diameter) coupled to a 2.45 N load cell. All measurements were performed with 5 samples for each scaffold type within a deformation interval of 10% of the initial scaffold height, executing linear compression with 0.15 mm/s, cyclic compression with physiological frequencies of 0.2 Hz and 2 Hz and a high frequency of 20 Hz, and instantaneous compression with a square function (0.2 Hz, *n* = 3). The apparent elastic modulus, the dissipation factor, and the relaxation time were determined. Measurement inaccuracies were prescribed by error propagation.

### 2.4. Bone Morphogenetic Protein 2 (BMP–2) Loading and Release

To perform a cytokine release study, the optimal rehydration volume of the scaffolds was determined to be 60 µL. Using a stock solution containing 83.3 ng/µL rhBMP–2 (recombinant human bone morphogenetic protein 2), each freeze-dried scaffold was soaked with the objective to be loaded with 5 µg of rhBMP-2. After loading the scaffolds, they were immediately transferred into Protein LoBind sealable vials (Eppendorf; Hamburg, Germany) to perform a release study. The BMP–2 loaded scaffolds were subjected to lixiviation for 28 days in sterile PBS. Anisotropic and isotropic scaffolds (*p* = 5 each) were maintained in 0.5 mL PBS solution for each day until the next measuring point. The lixiviation was carried out at 37 °C under shaking at 25 rpm. The supernatant was captured and replaced on the days 1, 2, 3, 4, 5, 7, 9, 11, 14, 17, 21, and 28. The release was investigated within 28 days using a Human BMP–2 ELISA Kit (Ray Biotech; Norcross, GA, USA) by measuring the exchanged buffer samples. 

### 2.5. Nonunion In Vivo Model 

All animal experiments were approved by the Institutional Animal Care and Use Committee of the Universidad de Navarra [Comité de Ética para la Experimentación Animal, CEEA #075-12(033-12)]. Twenty-seven 10 to 12-week-old Sprague–Dawley rats underwent surgery to create a critical-size segmental defect in the right femur. Animals were intubated and anesthetized with isofluorane. The hair over the right hind limb was clipped, and the area prepped with povidone iodine. Using a lateral approach, the right femoral shaft was exposed. The femur was then stabilized using an aluminum plate (20 mm long × 4 mm wide × 2 mm high) and four steel screws (1.5 mm diameter, 8 mm long). Using a bur, a critical size defect of about 4 mm was created in the middle of the diaphysis under continuous saline irrigation. A total of four groups were evaluated: anisotropic scaffold loaded with water (ANI, *p* = 7), isotropic scaffold loaded with water (ISO, *p* = 7), anisotropic scaffold loaded with 5 μg of rhBMP–2 (ANI–BMP2, *p* = 7), and isotropic scaffold loaded with 5 μg rhBMP–2 (ISO–BMP2, *p* = 6). After carefully introducing the corresponding scaffold/sponge in the gap of the femur, the muscles were carefully repositioned and the skin sutured. After recovery, the rats were treated with buprenorphine for three days and allowed unrestricted activity until sacrifice. After 10 weeks, the animals were sacrificed and both femurs, surgically treated and contralateral, were carefully removed and cleaned of all the soft tissue. Femurs were washed and kept in PBS at 4 °C until needed.

### 2.6. Radiography

For the repair process follow-up, X-rays were taken at weeks 1, 3, 5, 7, and 10 after the surgery using a cabinet X-ray, 20 s at 30kV (Faxitron Bioptics, Tucson, AZ, USA). The radiographic evaluation of the defect region was performed by three observers blindfolded at 5, 7, and 10 weeks through simplified radiographic union scoring tibia (RUST), single plane radiographs were classified into three healing categories. A complete, continuous cortical bone represented the criterion for the highest category (score = 2). The presence of a fracture line or a discontinuity in the callus of a defect led to a categorization in the intermediate category (score = 1). When the defect healing did not differ largely from the empty control, since no bone was formed to bridge the defect, the lowest category was chosen (score = 0). 

### 2.7. Micro computed Tomography (µCT)

The femora were scanned with a Siemens microCAT II scanner (Siemens Health care Gmbh, Munich, Germany) at 75 kVp and 250 μA, with an exposure time of 1250 ms. Using the AMIRA software for reconstruction, the noise was filtered and the data calibrated and segmented at a threshold of 1200 Hounsfield Units, differentiating between low mineralized, soft tissue (<1200), and high mineralized tissue (>1200). An evaluation of the quality of healing was carried out from µCT reconstructions by extracting two radiographic planes and quantifying the number of continuous cortices (maximum = 4, complete healing; minimum = 0, no healing).

### 2.8. Torsional Biomechanical Testing

After µCT analysis, two femurs of each group were randomly derived to histological analysis, while remaining samples were derived for mechanical testing. Both the repaired and the contralateral femora were evaluated mechanically for each rat. The mechanical test was performed using an Instron 8874 (Instron, Norwood, MA, USA). Both ends of the femurs were placed in custom-made molds and embedded with resin (Demotec–70). This resin was chosen because it does not reach high temperatures during curing. During mechanical testing, one femur end (metaphysis) remained fixed, whilst the other rotated in the internal direction at a speed of 0.1 degrees per s until ultimate failure. Pure torsion was ensured, as the load placement was set to zero and controlled, so neither traction nor compression was placed upon the bones. The femurs were kept moist during the whole procedure by wrapping them in soaked gauze. From the test, the maximum torque and the angle of failure were recorded. The repair rate was computed as the maximum torque displayed by the treated femur, relative to its control. In addition, the resistance to torsion shear stress and shear modulus of each femur of the groups containing BMP–2 was computed.

### 2.9. Histology

After CT analysis, selected femurs were fixed for 48 h with 10% buffered formalin (Panreac). Samples were decalcified using 10% EDTA–PVP solution (0.1 M Tris HCl, 10% EDTA, 7.5% polyvynilpirrolidone (PVP), pH 6.95), dehydrated in graded ethanol and xylene, embedded in paraffin, and sectioned at a thickness of 4 micrometers. The samples were serially sectioned and stained with Hematoxylin and Eosine (H and E) to visualize the center of the femur. 

### 2.10. Statistical Analysis

Unless otherwise indicated, graphic data are presented as mean ± SEM. For the biomechanical and morphometric graphic, data are presented as floating bars with whiskers (minimum to maximum). Statistical analysis was performed using GraphPad Prism 5.0 software (GraphPad Software Inc., La Jolla, CA, USA). The Kolmogorov–Smirnov test was applied to determine if the data presented normal distributions. When normal distribution was present, an analysis of variance (ANOVA) test followed by Tukey’s multiple comparisons test was performed to compare between the columns. Unpaired, two-tailed Student t-test was also performed for single pair group comparisons. When normal distribution was not present, and for RUST scoring data, a Kruskal–Wallis test followed by Dunnett’s multiple comparisons test was performed to compare between columns. The statistical significance was set at a *p* < 0.05.

## 3. Results

### 3.1. Cryostructured Scaffolds Characterization

**Figure 1:**
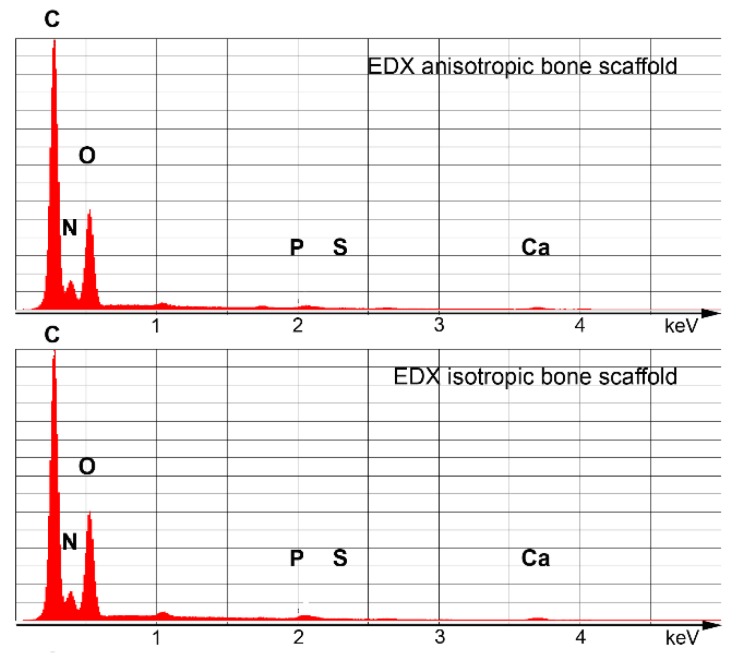
Energy Dispersive X-ray spectra of anisotropic and isotropic scaffolds.

The elementary composition of the resulting bone scaffolds was analyzed by Energy Dispersive X-ray spectroscopy (EDX). Due to the equivalent precursor compositions, the spectra of ANI and ISO scaffolds reached similar values of 40.4% Carbon, 11.2% Nitrogen, 23.6% Oxygen, and 23.6% Calcium for the dominant peaks, while 0.7% Phosphor and 0.4% Sulfur were detected for the minor peaks (Figure 1). 

**Figure 2:**
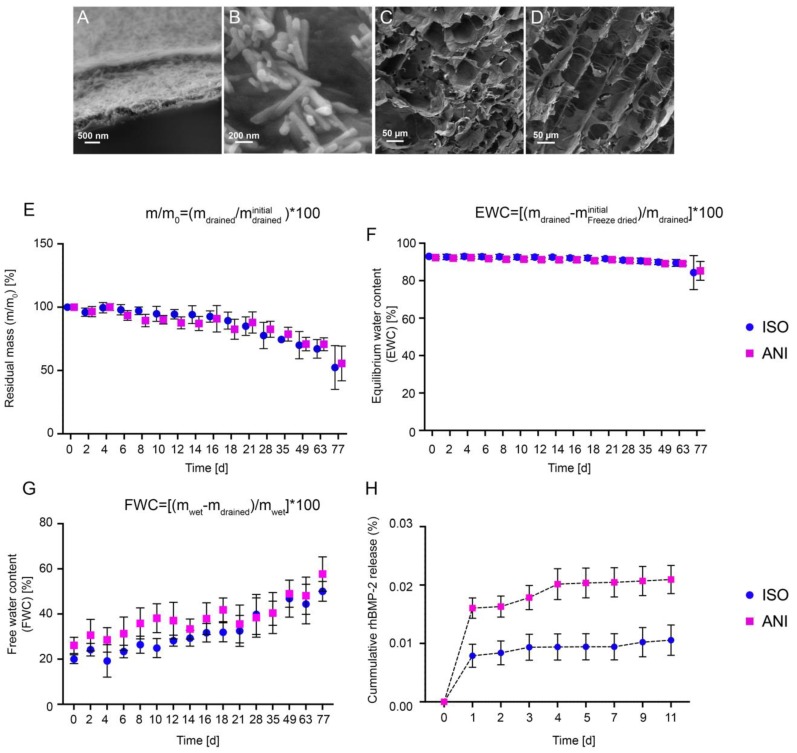
In vitro characterization of cryostructured bone scaffolds. (**A**–**D**) scanning electron microscope (SEM) images: (**A**) Cross-sectional image of typical lamellae presenting a homogeneous composite structure of collagen and hydroxyapatite; (**B**) Representative sub-micron hydroxyapatite crystals adhered to the inner surface of both scaffold types; (**C**) Cross section of the isotropic scaffolds (ISO); (**D**) Cross section of anisotropic pore structures (ANI); (**E**–**G**) Degradation behavior of the bone scaffolds under physiological conditions: (**E**) Residual mass (m/mo); (**F**) Equilibrium water content (EWC); (**G**) Free water content (FWC); (**H**) In vitro bone morphogenetic protein 2 (BMP–2) release from bone scaffolds. ISO, scaffold with isotropic pore structure; ANI, scaffold with anisotropic pore structure.

**Figure 3:**
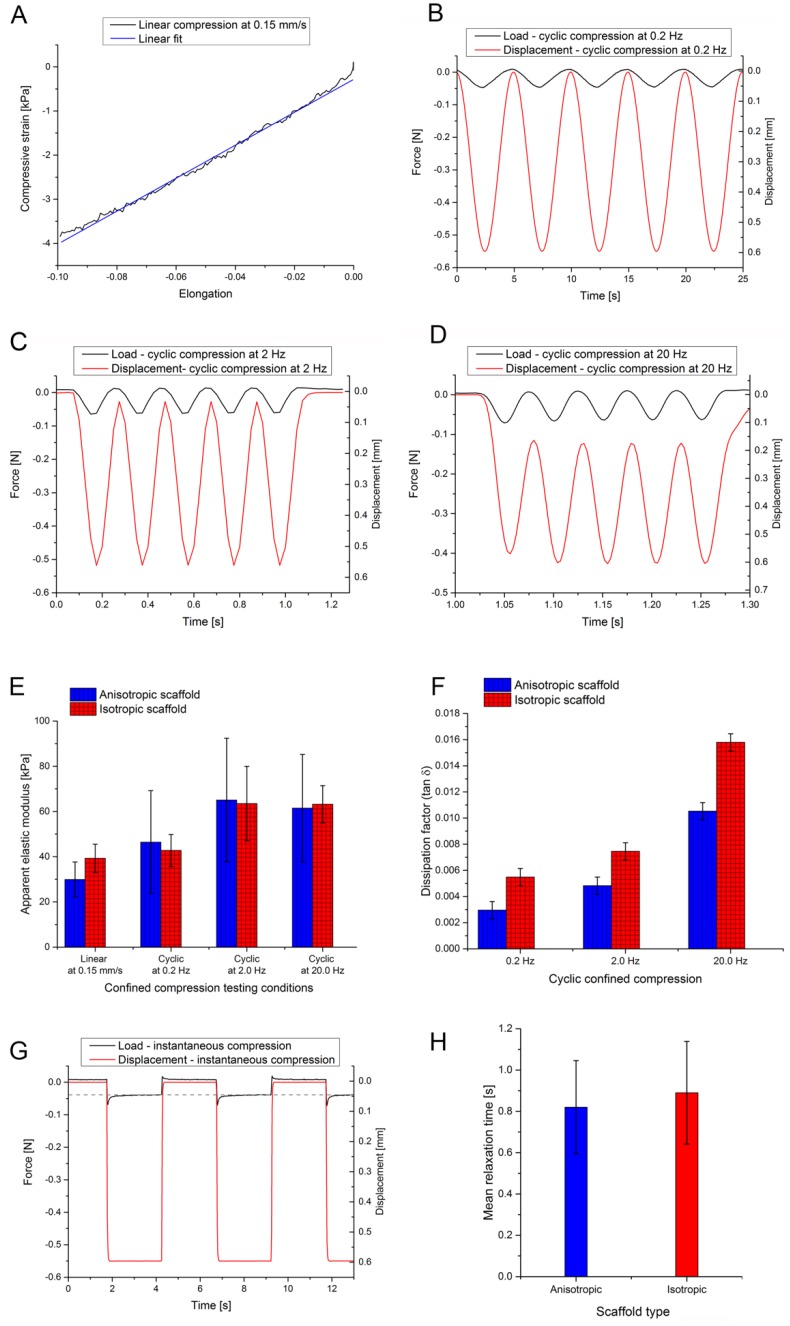
(**A**) Exemplary datasets of linear compression with a crosshead speed of 0.15 mm/s; (**B**–**D**) Cyclic compression with 0.2, 2, and 20 Hz for a bone scaffold with isotropic pore structure; (**E**) Resulting apparent elastic modulus for linear and cyclic compression; (**F**) Dissipation factor for linear and cyclic compression. Testing parameters: 0.15 mm/s, 0.2 Hz, 2 Hz, and 20 Hz respectively; error bars were derived by error propagation; (**G**) Exemplary dataset of instantaneous deformation by square function; (**H**) Mean relaxation time of tested scaffolds. Error bars represent standard deviation.

SEM images of the resulting bone scaffolds are depicted in Figure 2. A cross-section through a wall between the pores is presented in Figure 3A. The 0.5 µm thick walls consist of a homogeneous composite structure of collagen and hydroxyapatite. Here, a random distribution of the cuboid shaped hydroxyapatite crystals was present. Sub-micron hydroxyapatite crystals, which were adhered to the inner scaffold surface, were standing off the composite matrix structure, creating a huge surface area (Figure 2B). Cross-sections, perpendicular to the basic surface of the cylinder samples, revealed the inner pore structure in both types of scaffolds. While a random pore structure was present within the isotropic scaffolds (Figure 2C), the anisotropic scaffolds featured a homogeneously aligned pore structure (Figure 2D). The spongy, irregularly-shaped pores showed cross section widths of 88 ± 35 µm in diameter and were interconnected by randomly distributed and sized holes inside the pore walls (Figure 2C). Within the channel pores, pore widths of 65 ± 25 µm were supported by collagenous fiber pillars (Figure 2D).

The degradation behavior of the scaffolds was analyzed under physiological conditions. Over a time-span of 11 weeks, the scaffolds were evaluated in terms of change in residual mass, equilibrium water content, and free water content. The residual mass (m/mo) was determined as the relation between current and initial drained mass. Within the first 10 weeks, all scaffolds behaved similarly and showed a daily mass loss of 0.57% (Figure 2E). The equilibrium water content (EWC) was calculated as the relation of water still bound after draining to the drained weight of the scaffolds. Again, all scaffolds behaved nearly the same and showed an EWC loss of 0.066% per day within weeks 1 to 10 (Figure 2F). Additionally, the free water content (FWC) was determined as the relation of loosely adhered water to the wet weight of the bone scaffolds. On average, the FWC increased by about 0.38% per day. Due to the anisotropic channel pores, the anisotropic scaffolds released the loosely stored water more easily than the isotropic scaffolds (Figure 2G).

A release study of the BMP–2 loaded scaffolds was also performed. Each type of scaffold was loaded with 5 µg of rhBMP–2, and the resulting BMP–2 release was determined. Only minimal amounts, in the ng range, were released within the first two weeks. After this timespan, the released cytokine was below the detection limit of the kit (Figure 2H). Based on the cumulative release, the retention of the BMP–2 was calculated by assuming a successful initial loading of 5 µg rhBMP–2 for each scaffold. In summary, the BMP–2 release accounted for 0.022% of the isotropic scaffolds (ISO) and 0.043% of the anisotropic scaffolds (ANI). 

### 3.2. In Vivo Assessment of Cryostructured Scaffolds 

**Figure 4:**
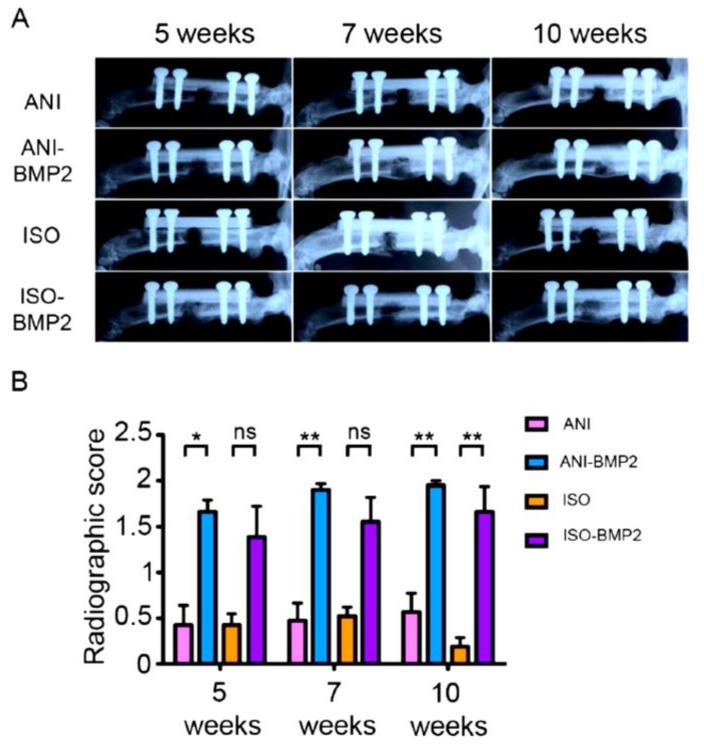
Radiographic follow-up of the reparative process. (**A**) Single plane radiographs were taken at 5, 7, and 10 weeks post-surgery. Representative samples are depicted for each group; (**B**) The healing was scored with a simplified RUST scoring system. *, *p* < 0.05; **, *p* < 0.01; ns, non-significant by Kruskal–Wallis test (*p* = 0.0001) followed by post-hoc Dunnett’s multiple comparisons test.

The biological evaluation of the bone scaffold performance was carried out in vivo by using the scaffolds to fill a critical size defect in the femurs of Sprague–Dawley rats. The in vivo groups of the study (each *p* = 6–7) contained either bone scaffolds or BMP–2 loaded (5 µg of rhBMP–2) bone scaffolds. The healing progression was monitored radiographically at weeks 1 (not shown), 3 (not shown), 5, 7, and 10 after the surgery (Figure 4A). Interestingly, all scaffolds containing rhBMP–2 showed radio-opacity from week 5 up to week 10, as scored by RUST. While all of the anisotropic scaffolds, supplemented with BMP–2, showed consistently high RUST scores from week 5 on, the isotropic rhBMP–2 containing scaffolds showed a lower RUST score, but without significant differences (Figure 4B). In the case of the pure scaffold groups, no bridging of the defect was observed, and a fracture line could be observed through the evaluation period. 

**Figure 5:**
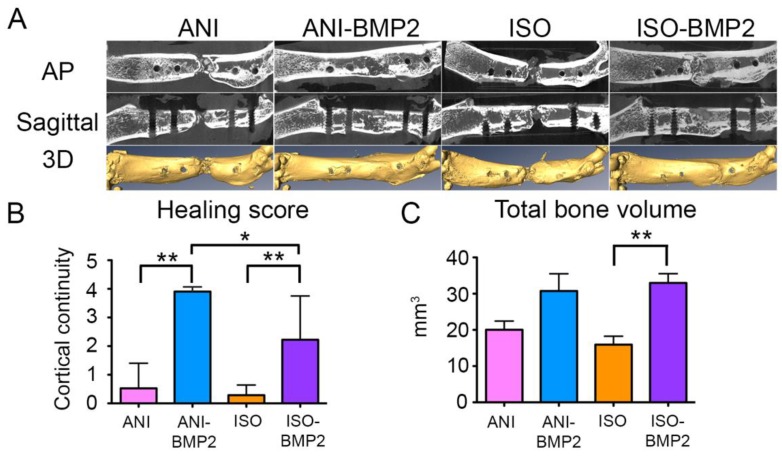
Micro computed tomography analysis of the reparative process. (**A**) Summary of the 3D reconstruction and its renderings. AP, apical plane; Sagittal, sagittal plane; (**B**) The cortical continuity of the samples was analyzed in two projections and quantified. *, *p* < 0.05; **, *p* < 0.01 by Kruskal–Wallis test (*p* = 0.0001) and post-hoc Dunnett’s test; (**C**) The total bone volume was quantified from the 3D reconstructions. **, *p* < 0.01 by one-way ANOVA (*p* = 0.0205) and post-hoc Tukey’s multiple comparisons test.

Representative µCT images of the explanted femurs confirmed the findings of the radiographic follow-up (Figure 5A). In agreement with the RUST evaluation, the best regeneration results, calculated as the number of continuous cortices in two radiographic planes, were visible for the BMP–laden anisotropic scaffolds (ANI–BMP2), while the isotropic scaffolds loaded with BMP–2 (ISO–BMP2) showed inferior, but still good, results (Figure 5B). An equivalent but more intense manifestation of this trend may be observed for the pure scaffolds. In most of the cases, an incomplete bridging of the defect was present for the anisotropic scaffolds, but only poor bone growth could be found in the case of the isotropic scaffolds. Within the final healing evaluation of the explants, one defect that was treated with an anisotropic scaffold even healed without the application of BMP–2 (data not shown). After 3D reconstruction, the bone volume in the femoral defect was calculated. Both scaffolds showed increased bone volume (Figure 5C) in the treated defect, being significant between the ISO and ISO–BMP2 groups (*p* = 0.0005). 

### 3.3. Biomechanical Testing

**Figure 6:**
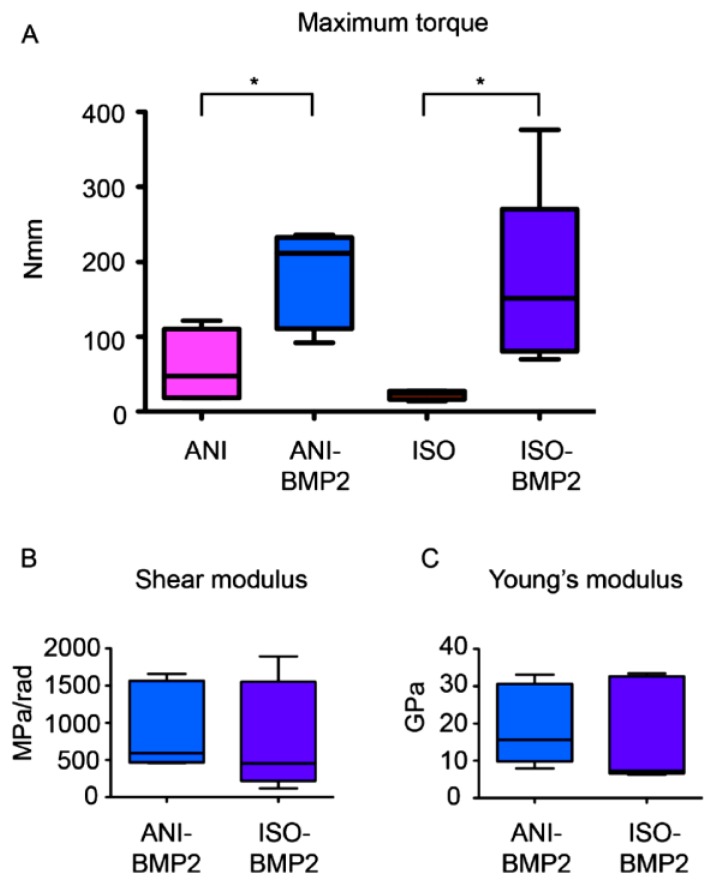
Determination of the biomechanical properties of the repaired bones. (**A**) Maximum torque of the different groups was determined. *, *p* < 0.05 by one Kruskal–Wallis test (*p* = 0.0129) and followed by Dunnett’s multiple comparisons test. No differences were found in shear modulus (**B**) and Young’s modulus (**C**) between anisotropic scaffolding bone morphogenetic protein 2 (ANI–BMP–2) and isotropic scaffolding (ISO)–BMP–2 groups.

**Figure 7:**
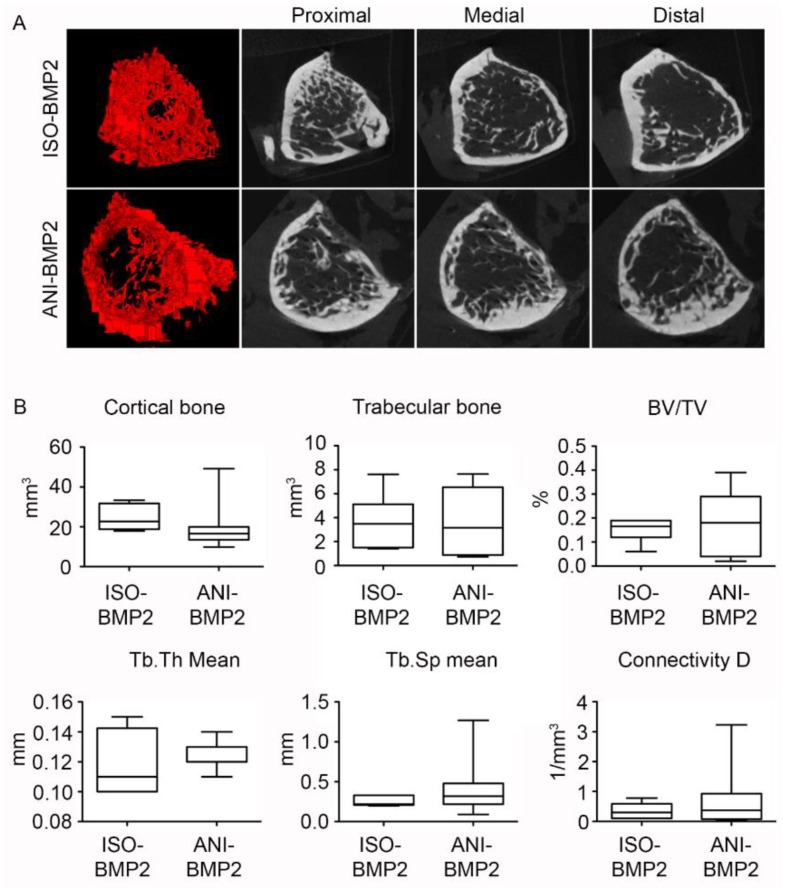
Similar properties of the newly synthesized bone in the animals treated with isotropic and anisotropic scaffolds loaded with bone morphogenetic protein 2 (BMP–2). (**A**) 3D reconstruction and single plane renderings from the proximal edge (left) to the distal edge (right) of the trabecular bone in the repair callus; (**B**) Morphometric measurements of the repaired calluses. BV/TV, bone fraction; Tb.Th, trabecular thickness; Tb.Sp, trabecular separation.

The mechanical characterization was performed by torsion and yielded the maximal torsional moment, as depicted in Figure 6. The defects treated with both types of BMP–2 loaded scaffolds attained nearly half of the mean torsional moment that was observed for intact femurs of the contralateral side. Without the employment of rhBMP–2, significantly lower values were obtained. While the application of the anisotropic scaffolds resulted in a mean torsional moment of one third of the ones that were exhibited by the use with BMP–2, the isotropic scaffolds only yielded one eighth of this value (Figure 6A). Since individual samples of native tissues tend to show a divergent behavior, the values obtained for the intact and repaired femurs showed considerably large individual variations. The comparison between the mechanical properties of ISO–BMP–2 and ANI–BMP–2 repaired bones did not show major differences (Figure 6B). In consequence, neither were any differences detected in the morphometric properties of the bone callus (Figure 7). 

### 3.4. Histological Analysis

**Figure 8:**
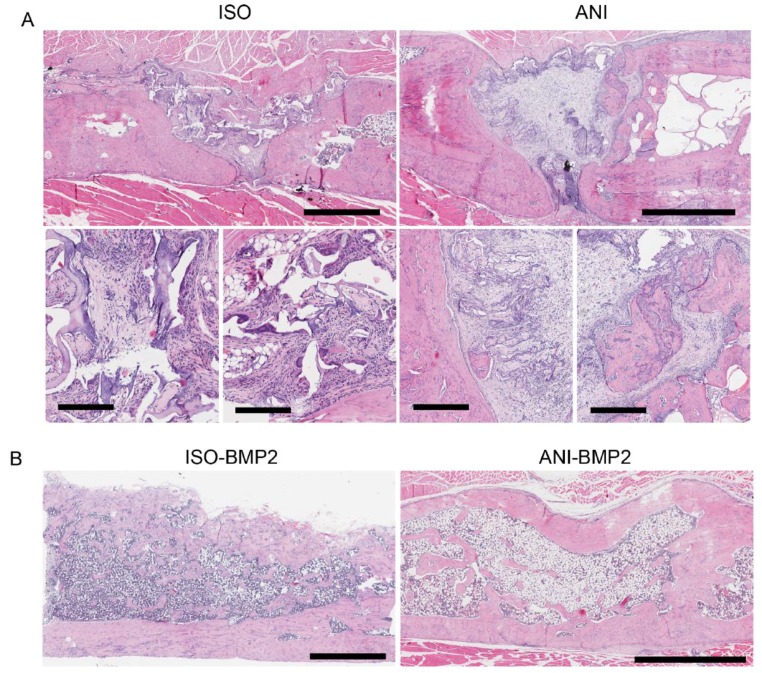
Histological findings. (**A**) Representative Haematoxylin and Eosin stain of samples treated with empty scaffolds. Scale bar upper panel: 1.5 mm isotropic scaffolding (ISO), 2 mm anisotropic scaffolding (ANI); scale bar lower panels: 450 µm. (**B**) Representative H and E stain of samples treated with bone morphogenetic protein 2 (BMP–2) laden scaffolds. Scale bars: 1.5 mm (ISO–BMP–2), 2 mm (ANI–BMP–2).

Histological examination confirmed the radiographic imaging. H and E stains showed that without rhBMP–2, only incomplete regeneration processes could be observed (Figure 8A). A better appearance was visible in the case of the anisotropic pore structure in terms of cellularity and the presence of bone islands, which suggested that pore orientation served as guidance for the migrating bone progenitor cells (Figure 8A, lower panels). For the rhBMP–2 loaded scaffolds, cortical bone and trabecular bone were generated. By comparison, it seems that more trabeculi and lacunas were formed inside the isotropic scaffolds. The anisotropic bone scaffolds still exhibited the highest overall healing quality at the cortical level, suggesting that anisotropic pore structures facilitate invasion/migration of progenitor cells from the edges of the defect.

## 4. Discussion

There is a wide use of BMPs in orthopedic trauma and tissue engineering, especially for the regeneration of bone, cartilage, and tendons [23,24,25]. For bone regeneration, their use is commonly associated with collagen sponges [10]. In fact, the combination of rhBMP–2 with collagen sponges is an FDA-approved treatment for long bone non-unions, open tibial fractures, and spinal fusions [11]. 

The major problem with the delivery of BMPs is the supraphysiological dosage that could result in heterotopic ossification, increased resorption, and inflammation [9,26,27]. Thus, several strategies have been proposed to increase the efficiency of BMP-based treatments, trying to reduce the bulk of BMPs added to the bone defects. One of these strategies is based on the optimization of BMP delivery, through changes in porosity and/or retention properties of the materials of a given scaffold or composite implant [11]. 

While, for bone regeneration, several strategies are centered in BMP–2 delivery, the function of the scaffold has been reduced to an early role, as cell growth support in the initial steps of regeneration [28]. In this scenario, to be optimal, a scaffold must present low immunogenicity and biodegradability, inducing a limited inflammatory response [11]. Here, out of type I collagen and hydroxyapatite precursors, we produced two types of cylindrical bone scaffolds, fitting to fill out critical size defects in rat femurs. Scaffolds with anisotropic channel pores, which propagate from one basis of the cylinder to the other (ANI), and scaffolds featuring sponge-like, isotropic pores, which are randomly distributed among the matrix (ISO), were fabricated. Our goal was to determine if the structure of the scaffold could influence the efficiency of rhBMP–2 treatment and delivery. Both bone scaffolds differed solely in pore structure, presenting similar composition, degradation, and rhBMP–2 release rate in vitro. In vivo, both scaffolds efficiently delivered rhBMP–2, allowing significant repair of critical size defects. Anisotropic scaffolds are more efficient in allowing full healing, suggesting that an anisotropic morphology enhances mesenchymal progenitor migration. No significant differences were detected in the mechanical properties, bone structure, and bone volume of the regenerated bone after treatment with rhBMP–2. Subtle differences were also noted when scaffolds were used without rhBMP–2, resulting in better performance of the ANI scaffolds, although the differences were not statistically significant.

The leaching out of biological factors is an important aspect in the efficiency of certain therapies. Although the presence BMPs is known to induce the formation of bone tissue, even in non-skeletal implantation sites, clinical results of BMP–2-loaded collagen or hydroxyapatite/tri-calcium phosphate scaffolds do not always show a significant increase in bone healing of critical-sized defects when compared to the scaffolds alone [29]. The fast release of rhBMP–2 was presumed to cause these unsatisfying results for the abovementioned scaffolds [29,30].

In contrast to the common opinion that a low release of BMPs over a long period of time does not lead to beneficial bone healing results, the regeneration of a critical sized bone defect could be demonstrated by using anisotropically structured collagen I/hydroxyapatite scaffolds with a very high BMP–2 retention capability. Calcium phosphates are known to bare a high binding potential of proteins like BMP-2 [30]. Due to the use of sub-micron hydroxyapatite crystals for the generation of cryostructured bone scaffolds, a high surface area with attractive BMP–2 binding sites has been created, so that after all, only less than 0.05% of the initially loaded cytokine is released within 4 weeks.

There is not a consensus on the right amount of implanted BMP–2 to induce efficient bone regeneration. In long-bone critical defects, a range between 2.25 µg and 11.25 μg of rhBMP–2 delivered in poly(lactic–co–glycolic acid) scaffolds demonstrated filled critical femoral defects without important side effects [9]. Others reported efficient void filling using between 1 µg to 5 μg of rhBMP–2 delivered from alginate [31]. Here, we used a bulk amount of rhBMP–2 of 5 μg, which seems sufficient for optimal bone regeneration. In any case, these amounts of rhBMP–2 are far from inducing the more important side effects, as demonstrated by ectopic implantation in mice muscle [32].

## 5. Conclusions

In conclusion, cryostructured anisotropic collagen scaffolds allowed an optimized delivery of low rhBMP–2 doses, improving bone regeneration by enhancing the recruitment of osteogenic progenitor cells.

## Supplementary Materials

The following are available online at https://www.mdpi.com/1996-1944/12/19/3105/s1, Figure S1: Temperature diagraphs of anisotropic and isotrophic scaffolds.
