# Supplementary material for: Anisotropic Cryostructured Collagen Scaffolds for Efficient Delivery of RhBMP–2 and Enhanced Bone Regeneration
Source: Materials (Basel). 2019 Sep 24;12(19):3105. doi: 10.3390/ma12193105 (PMC6804013; doi:10.3390/ma12193105)
Supplement: Supplementary file 1 [file materials-12-03105-s001.pdf]

materials

Article

Anisotropic Cryostructured Collagen Scaffolds for Efficient Delivery of RhBMP–2 and Enhanced Bone Regeneration

Kai Stuckensen [1], José M. Lamo-Espinosa [2], Emma Muiños-López [3], Purificación Ripalda-Cemboráin [2,3], Tania López-Martínez [3], Elena Iglesias [3], Gloria Abizanda [3], Ion Andreu [4], María Flandes-Iparraguirre [4], Juan Pons-Villanueva [2], Reyes Elizalde [4], Joachim Nickel [5], Andrea Ewald [1], Uwe Gbureck [1], Felipe Prósper [3,6], Jürgen Groll [1,*] and Froilán Granero-Moltó [2,3,*]

Supplementary Materials

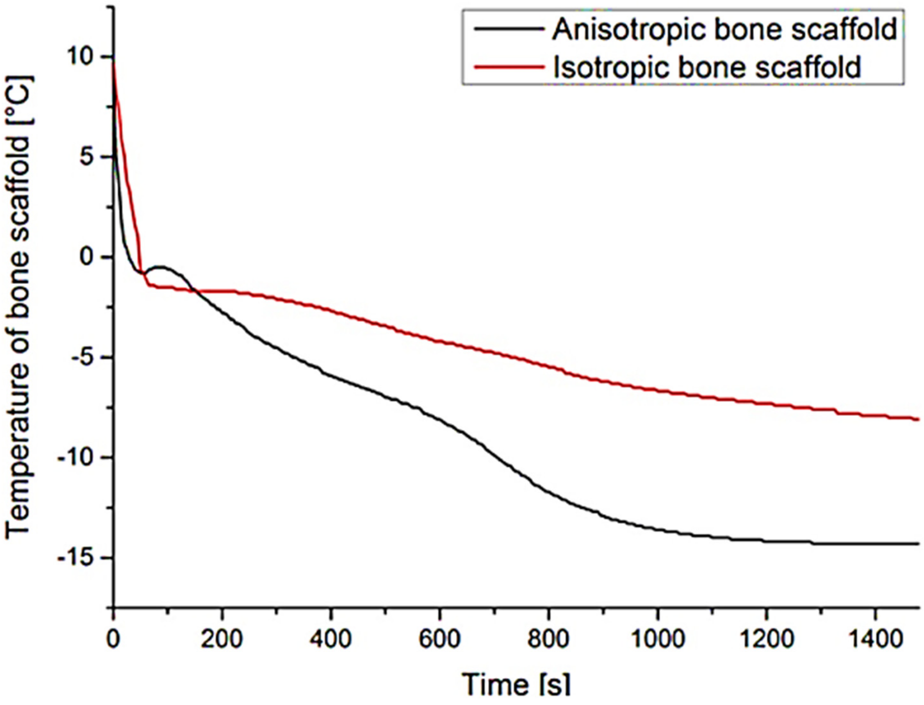

Figure S1. Temperature diagraphs of anisotropic and isotrophic scaffolds.
